# Association of Angiotensin-Converting Enzyme (ACE) Gene Single Nucleotide Polymorphisms (SNPs) With Hypertension in Older Japanese Adults: A Cross-Sectional Study Nested Within the Nagasaki Islands Study (NaIS)

**DOI:** 10.7759/cureus.82193

**Published:** 2025-04-13

**Authors:** Serina Koto, Kazuhiko Arima, Hiroki Nakashima, Ayuko Takatani, Satoshi Mizukami, Yuji Shimizu, Mami Tamai, Atsushi Kawakami, Koichiro Hamada, Takahiro Maeda, Shin-ya Kawashiri, Yasuhiro Nagata, Kiyoshi Aoyagi

**Affiliations:** 1 Department of Public Health, Nagasaki University Graduate School of Biomedical Sciences, Nagasaki, JPN; 2 Department of Immunology and Rheumatology, Nagasaki University Graduate School of Biomedical Sciences, Nagasaki, JPN; 3 Leading Medical Research Core Unit, Nagasaki University Graduate School of Biomedical Sciences, Nagasaki, JPN; 4 Epidemiology Section, Osaka Institute of Public Health, Osaka, JPN; 5 Department of General Medicine, Nagasaki University Graduate School of Biomedical Sciences, Nagasaki, JPN; 6 Department of Community Medicine, Nagasaki University Graduate School of Biomedical Sciences, Nagasaki, JPN; 7 Center for Collaborative Medical Education and Development, Nagasaki University Institute of Biomedical Sciences, Nagasaki, JPN

**Keywords:** angiotensin-converting enzyme (ace), genotype, hypertension, nagasaki islands study, single nucleotide polymorphisms (snps)

## Abstract

Aim

We aimed to investigate the association between single nucleotide polymorphisms (SNPs) in the angiotensin-converting enzyme (ACE) gene and hypertension in elderly Japanese individuals.

Methods

This cross-sectional validation study was nested within the Nagasaki Islands Study (NaIS), involving 1,766 community-dwelling participants aged 65 years and older. Height and weight were measured. Body mass index (BMI) was calculated from height and weight, and blood pressure was measured by trained staff. Antihypertensive medication use and lifestyle factors, including alcohol consumption, smoking habits, and exercise, were assessed by interviews. The SNP (rs4309) was genotyped using fluorescent hydrolysis probes from peripheral blood mononuclear cells.

Hypertension was defined as using antihypertensive medication or a systolic blood pressure of ≥140 mmHg and/or a diastolic blood pressure of ≥90 mmHg in those not on medication. Logistic regression analysis was used to calculate adjusted odds ratios (ORs) for hypertension, adjusting for potential confounders.

Results

Hypertension prevalence was higher in the CC/CT genotype group compared to the TT group (71.5% vs 66.4%, p=0.025). Logistic regression showed that the CC/CT genotype was independently associated with a higher likelihood of hypertension than the TT genotype (OR=1.25, 95% confidence interval (CI) 1.001-1.548) after adjusting for age, BMI, gender, alcohol consumption, smoking, and exercise.

Conclusion

The CC and CT genotypes of the ACE gene were independently associated with hypertension, regardless of age, BMI, gender, and lifestyle factors. These findings support a genetically informed approach to hypertension prevention.

## Introduction

Essential hypertension, commonly referred to as hypertension, is a prevalent and frequently occurring condition that is primarily characterized by elevated blood pressure and is the leading cause of death related to cardiovascular disease [1]. The global prevalence of hypertension is projected to reach 20.3% (20.2%-20.4%) by 2040; therefore, it is a significant public health issue [2]. Among individuals older than 80 years of age, the prevalence of hypertension, which increases with age, is 74% [3]. Therefore, hypertension prevention is a critical concern in Japan because of its aging population.

The angiotensin-converting enzyme (ACE) gene plays a key role in the renin-angiotensin system, which regulates blood pressure. Single nucleotide polymorphisms (SNPs) in the ACE gene have been studied in relation to hypertension, and regional differences in susceptibility have been revealed [4,5].

The ACE gene polymorphisms rs7213516, rs7214530, and rs4290 - commonly observed in African Americans, a population with a higher risk of hypertension [6] and reduced responsiveness to ACE inhibitors [7,8] - have been associated with decreased ACE mRNA expression [9]. These three SNPs are promoter variants located upstream of the transcription start site. In addition, the SNP rs1800764, situated upstream and near the ACE promoter, has been significantly associated with both ACE activity and early-onset hypertension [10]. These findings suggest that SNPs located in or near the ACE gene promoter may influence ACE activity through modulation of mRNA expression, thereby contributing to hypertension susceptibility.

Studies performed in China have indicated that an effective model of predicting the risk of essential hypertension is the combined effect of ACE gene SNPs rs4291, rs4309, and rs4461142 [4]. This finding led us to explore whether SNP rs4309 in the ACE gene is similarly associated with hypertension in older Japanese community-dwelling individuals. 

We developed a registry that includes blood pressure data as part of the Nagasaki Islands Study (NaIS) [6] conducted in Goto City, Nagasaki Prefecture. The NaIS aims to monitor diseases considered to be influenced by lifestyles in the community. Using the data from the NaIS, we investigated the association between rs4309 and hypertension.

## Materials and methods

This cross-sectional study included residents of Goto City, Nagasaki Prefecture, who were 65 years of age or older, participated in specific health examinations between 2017 and 2019, and provided informed consent for participation. Individuals with missing data were excluded from the analysis. This study was approved by the Ethics Committee of the Nagasaki University Graduate School of Biomedical Sciences (no. 14051404-12).

Based on the recommendation by Peduzzi et al. [11], which states that logistic regression requires at least 10 events per explanatory variable to ensure reliable and stable estimates, a minimum of 70 events was required for analysis, as seven explanatory variables were included in the model.

Anthropometric measurements were performed to collect height and weight data, with participants wearing light clothing, using an automatic body composition analyzer (BF-220; Tanita, Itabashi, Tokyo, Japan). Body mass index (BMI) was then calculated. Blood pressure was measured on the participants’ arms in a seated position using an automatic sphygmomanometer (HEM-907; Omron, Kyoto, Japan). If the blood pressure readings were excessively high or considered unreliable, measurements were repeated or taken manually [12]. Information regarding age, sex, antihypertensive medication use, excessive alcohol consumption, smoking habits, and exercise habits was collected via interviews. Hypertension was defined as the use of antihypertensive medication, systolic blood pressure ≥140 mmHg, or diastolic blood pressure ≥90 mmHg. Excessive alcohol consumption was defined as daily intake of ≥40 g for men and ≥20 g for women. Exercise habits were defined as engaging in light sweat-inducing exercise for at least 30 minutes per day at least two days per week for at least one year. Peripheral blood mononuclear cells were collected from the participants and analyzed using SNP genotyping and real-time polymerase chain reaction with fluorescent hydrolysis probes (TaqMan probe method, assay ID: C___1247706_10; the call rate was 94.9% in this assay).

Participants were grouped according to sex and ACE genotype and compared. We used the Mann-Whitney U test to compare continuous data of two groups and the Kruskal-Wallis test to compare continuous data of three groups. Categorical data were compared using the chi-squared test.

To investigate the effects of ACE gene polymorphisms on hypertension, we conducted a logistic regression analysis with hypertension as the dependent variable and adjusted for age, BMI, sex, excessive alcohol intake, smoking habits, and exercise habits. We calculated the odds ratios (ORs) and 95% confidence intervals (CIs). P value <0.05 was considered statistically significant. All statistical analyses were performed using IBM SPSS Statistics version 30.0.0.0 (IBM Corp., Armonk, USA). 

## Results

Table 1 presents the characteristics of the study participants (653 male and 1,113 female participants; a total of 1,766 participants) by sex. The height, weight, BMI, excessive alcohol consumption rate, and smoking rate of men were significantly higher than those of women. Hypertension rates of men and women were 71.1% and 68.7%, respectively, indicating no significant differences between groups.

**Table 1:** Characteristics of study participants according to sex ACE, angiotensin-converting enzyme gene; BMI, body mass index; DBP, diastolic blood pressure; Q1, first quartile; Q3, third quartile; SBP, systolic blood pressure.

	Male (n=653)	Female (n=1113)	P value
	Median (Q1-Q3)	Median (Q1-Q3)	
Age (years)	73.0 (69.0-79.0)	74.0 (69.0-79.0)	0.749
Height (cm)	162.8 (158.4-167.0)	150.5 (146.8-154.0)	<0.001
Weight (kg)	61.7 (55.0-68.1)	50.4 (45.1-56.4)	<0.001
BMI (kg/m^2^)	23.2 (21.4-25.2)	22.4 (20.2-24.8)	<0.001
SBP (mmHg)	136.0 (124.0-148.0)	137.0 (125.0-150.0)	0.142
DBP (mmHg)	76.0 (68.0-85.0)	76.0 (68.0-84.0)	0.747
	n (%)	n (%)	
ACE genotype (rs4309)			0.654
CC (minor/minor)	130 (19.9)	202 (18.1)	
CT (major/minor)	283 (43.3)	490 (44.0)	
TT (major/major)	240 (36.8)	421 (37.8)	
Hypertension	464 (71.1)	765 (68.7)	0.309
Antihypertensive medication use	347 (53.1)	520 (46.7)	0.009
Excessive alcohol consumption	27 (4.1)	13 (1.2)	<0.001
Current smoking	109 (16.7)	18 (1.6)	<0.001
Exercise	355 (54.4)	553 (49.7)	0.061

The ACE gene rs4309 genotype was classified into three types: CC (minor/minor) (18.8%), CT (minor/major) (43.8%), and TT (major/major) (37.4%). The prevalence of hypertension and distribution of ACE gene genotypes did not significantly differ between men and women. 

The background characteristics of the participants were examined for each ACE gene genotype (CC, CT, and TT) (see Table 4 in Appendices). The hypertension rates of the CC, CT, and TT groups were 70.5%, 71.9%, and 66.4%, respectively; these were not significantly different. Similarly, the antihypertensive medication use rates were 50.6%, 52.1%, and 44.8% for the CC, CT, and TT groups, respectively. The hypertension and antihypertensive medication use rates of the TT group were the lowest among the three groups.

A logistic regression analysis was conducted using hypertension as the dependent variable. The ORs for CT and CC were calculated using TT as a reference (see Table 5 in Appendices). The Hosmer-Lemeshow test was performed to confirm that the model fit the data, with p values of 0.071 and 0.093 for model 1 and model 2, respectively. In the model adjusted for age, BMI, sex, and lifestyle factors (model 2), the adjusted OR for hypertension among individuals with the CT genotype was 1.301 (95% CI, 1.028-1.648), indicating that CT is an independent risk factor for hypertension. In contrast, the adjusted OR for hypertension among individuals with the CC genotype was 1.122 (95% CI, 0.831-1.515). Although the relationship between the number of C alleles and hypertension risk was not linear, the presence of the C allele tended to be associated with an increased risk of hypertension. Based on this trend, individuals with the CT and CC genotypes were combined into a single group (the CT and CC group) for further analyses. 

Table 2 presents the characteristics of the study participants stratified by ACE gene genotypes (CC and CT vs. TT). The prevalence of hypertension and antihypertensive medication use in the CC and CT group was significantly higher than that of the TT group. However, no significant differences in systolic blood pressure or diastolic blood pressure were observed. No significant differences in sex distribution, excessive alcohol consumption, smoking status, or exercise habits were observed.

**Table 2:** Characteristics of study participants according to ACE genotypes ACE, angiotensin-converting enzyme gene; BMI, body mass index; DBP, diastolic blood pressure; Q1, first quartile; Q3, third quartile; SBP, systolic blood pressure.

	CC and CT (n=1105)	TT (n=661)	P value
	Median (Q1-Q3)	Median (Q1-Q3)	
Age (years)	74.0 (69.0-79.0)	73.0 (69.0-79.0)	0.166
Height (cm)	153.9 (148.7-160.5)	154.1 (149.3-160.7)	0.404
Weight (kg)	53.9 (48.0-62.1)	53.8 (47.8-61.3)	0.85
BMI (kg/m^2^)	22.8 (20.6-24.9)	22.7 (20.6-25.0)	0.603
SBP (mmHg)	136.0 (124.0-149.0)	137.0 (125.0-149.0)	0.96
DBP (mmHg)	76.0 (68.0-84.0)	76.0 (68.0-84.0)	0.82
	n (%)	n (%)	
Hypertension	790 (71.5)	439 (66.4)	0.025
Antihypertensive medication use	571 (51.7)	296 (44.8)	0.005
Sex (male)	413 (37.4)	240 (36.3)	0.653
Excessive alcohol consumption	24 (2.2)	16 (2.4)	0.734
Current smoking	71 (6.4)	56 (8.5)	0.107
Exercise	570 (51.6)	338 (51.1)	0.855

Table 3 presents the results of the logistic regression analysis with hypertension as the dependent variable. Using TT as a reference, the OR for the CT and CC group was calculated.

**Table 3:** Multivariable analysis results according to ACE genotypes ACE, angiotensin-converting enzyme gene; BMI, body mass index; CI, confidence interval; OR, odds ratio.

	Unit	Crude	Model 1	Model 2
		OR (95% CI)	OR (95% CI)	OR (95% CI)
ACE genotype				
TT	Reference	1	1	1
CC and CT	Reference	1.268 (1.030-1.561)	1.246 (1.003-1.549)	1.245 (1.001-1.548)
Age	+1 year		1.073 (1.054-1.092)	1.075 (1.056-1.094)
BMI	+1 kg/m²		1.177 (1.136-1.220)	1.176 (1.135-1.218)
Sex	Female/male		1.032 (0.826-1.290)	1.025 (0.810-1.297)
Excessive alcohol consumption	Yes/no			2.352 (1.046-5.288)
Current smoking	Yes/no			0.859 (0.566-1.305)
Exercise	Yes/no			0.922 (0.745-1.142)

The Hosmer-Lemeshow test was performed to confirm that the model fit the data, yielding p values of 0.349 and 0.056 for model 1 and model 2, respectively. In the model adjusted for age, BMI, and sex (model 1), the adjusted OR for hypertension among the CC and CT group was 1.246 (95% CI, 1.003-1.549). After further adjustment for lifestyle factors (model 2), the adjusted OR was 1.245 (95% CI, 1.001-1.548). These findings suggest that the minor allele of rs4309 is an independent risk factor for hypertension among older Japanese individuals, irrespective of covariates.

To confirm the absence of sex-related trends, sensitivity stratified by sex was analyzed. For men, a logistic regression analysis with hypertension as the dependent variable was performed (see Table 6 in Appendices). Using TT as a reference, the OR for hypertension among the CT and CC group was calculated. The Hosmer-Lemeshow test confirmed that the model fit the data, with p values of 0.881 and 0.186 for model 1 and model 2, respectively.

A similar analysis was conducted for women (see Table 7 in Appendices). The Hosmer-Lemeshow test confirmed that the model fit the data, with p values of 0.688 and 0.131 for model 1 and model 2, respectively.

In the logistic regression analysis adjusted for age, BMI, and lifestyle factors, the adjusted ORs for hypertension associated with the minor allele of rs4309 were 1.265 (95% CI, 0.881-1.815) for men and 1.220 (95% CI, 0.927-1.606) for women. These findings indicated the absence of sex-related differences in the trends observed during this study.

## Discussion

Our study demonstrated that the minor allele of the ACE gene rs4309 is an independent risk factor for hypertension in older Japanese community-dwelling individuals. Age, BMI, and excessive alcohol consumption were positively associated with hypertension.

The renin-angiotensin-aldosterone system is an important mechanism that maintains hemodynamic stability by regulating extracellular fluid volume, sodium balance, and cardiovascular function [13]. The main enzyme in the renin-angiotensin-aldosterone system is ACE; ACE converts angiotensin I into angiotensin II, which is a powerful vasoconstrictor. Angiotensin II binds to angiotensin-1 receptors, thus causing a powerful vasoconstrictive effect and increasing blood pressure. ACE inhibitors prevent the production of angiotensin II by inhibiting ACE; therefore, they have an antihypertensive effect [14]. ACE inhibitors are widely used to treat hypertension, and ACE is an important target for hypertension treatment.

ACE gene polymorphisms and hypertension

Renin-angiotensin-aldosterone system overactivity has been linked to the development of hypertension [13]. Specific diseases include renovascular hypertension, in which renin is produced because of decreased blood pressure in the glomerular arterioles, thus causing increased blood pressure, and primary aldosteronism, in which blood pressure increases because of excessive secretion of aldosterone from the adrenal glands. Because of the role of the renin-angiotensin system in blood pressure regulation, ACE is a strong risk factor for essential hypertension [1].

Several SNPs in the ACE gene have been linked to hypertension. For example, a study of Korean patients revealed that the OR for hypertension was 2.11 for those with the GG genotype for rs4343 [15]. Additionally, a study conducted in Mexico found that ACE gene polymorphisms rs4291, rs4335, rs4344, rs4353, rs4362, and rs4363 were independently associated with hypertension, independent of other hypertension-related risk factors [16].

Based on these findings, it may be possible to identify high-risk individuals for hypertension using ACE gene SNPs and to prevent or delay the onset of hypertension by providing targeted lifestyle interventions from a younger age. Furthermore, previous studies have reported associations between ACE gene SNPs and responsiveness to ACE inhibitors [7,8], suggesting that genotyping of these SNPs could enable the selection of more effective antihypertensive medications tailored to individual patients.

ACE gene rs4309 polymorphism

rs4309 has been investigated to determine its association with essential hypertension [4], Alzheimer’s disease [17,18], aspirin asthma [19], systemic lupus erythematosus [20], systemic sclerosis [21], and diabetic nephropathy in patients with type 1 diabetes [22]. Some studies have reported an association between the rs4309 SNP and essential hypertension; however, consistent results have not yet been reported. For example, a study in China suggested that a combined model comprising rs4291, rs4309, and rs4461142 was a predictor of essential hypertension. However, the frequency of rs4309 polymorphism in the hypertensive group was not significantly different from that in the control group [4]. Therefore, the role of rs4309 in hypertension has not been fully elucidated.

rs4309 is a nonsynonymous mutation in the exon region. Nonsynonymous mutations do not involve amino acid substitutions, but they can affect splicing and are involved in carcinogenesis [23]. We did not find any reports of studies that examined the effect of rs4309 on splicing; however, it is possible that rs4309 had some effect on the development of hypertension, which could explain the results of this study.

Comparison with previous studies

During our study, the minor allele of the ACE gene rs4309 was identified as a risk factor for hypertension among older Japanese community-dwelling individuals. To the best of our knowledge, no previous studies have examined the relationship between rs4309 and hypertension in the Japanese population.

Wang et al. [4] conducted a study of 400 Han Chinese patients with hypertension and 100 healthy control participants in Anhui Province, China, and concluded that a combined model including rs4309 was a predictor of essential hypertension but did not recognize rs4309 as an independent risk factor for hypertension.

We identified four possible reasons for the discrepancies between our results and those reported by Wang et al. [4]. First, allele frequencies in the study populations were different. The study by Wang et al. [4] found the following genotype distributions of rs4309: CC, 11.4%; CT, 46.4%; and TT, 42.2%. However, in this study, the distributions were as follows: CC, 18.8%; CT, 43.8%; and TT, 37.4%.

In this study, the population had a high frequency of the risk allele C; therefore, the power of this study may have been increased. Second, the number of participants in the target groups differed. Wang et al. [4] included 500 study participants; however, the present study included 1,766 participants, which may have increased the detection power of this study. Third, the ages of the target populations differed. Wang et al. [4] included study participants who were 18 years or older, and the average ages of those in the hypertension group and the control group were 68.0 years and 63.7 years, respectively. In this study, however, the study participants were 65 years or older, and the median ages of men and women were 73.0 years and 74.0 years, respectively. The population in the present study was older than that included in the study by Wang et al.; therefore, it is possible that the effects of SNPs and lifestyle factors accumulated and manifested as hypertension. Fourth, our target population was homogeneous. The city of Goto in Nagasaki Prefecture, which was the target location of this study, is located on a remote island and residents rarely travel from the island to other places; therefore, the diet, climate, and customs of the residents are relatively uniform. By targeting a highly homogeneous population, the effects of SNPs can be detected with high sensitivity.

Age and hypertension

In our study, age was identified as a risk factor for hypertension among older Japanese individuals. Another study reported that older individuals are more susceptible to hypertension because the amount of Klotho protein in their blood decreases with age, thus activating the pathway for blood vessel constriction when they consume salt; this mechanism can cause decreased renal blood flow and increased blood pressure [24]. Our results did not contradict those findings.

BMI and hypertension

In our study, BMI was identified as a risk factor for hypertension among older Japanese individuals. A study of mice indicated that one mechanism that links hypertension and obesity is insulin receptor substrate-1 deficiency, which causes insulin resistance in muscle and adipose tissue; however, in the kidney, insulin action is mediated by insulin receptor substrate-2, and it is considered the mechanism by which high insulin levels cause sodium retention [25]. Our results did not contradict those findings.

Excessive alcohol consumption and hypertension

In our study, excessive alcohol consumption was identified as a risk factor for hypertension in older Japanese individuals. Another study reported that excessive alcohol consumption (three or more drinks per day) significantly increases the blood pressure of individuals with and without hypertension and is strongly associated with hypertension [26]. The results of this study did not contradict those findings.

The specific physiological relationship between excessive alcohol consumption and hypertension is not clear; however, factors that contribute to this relationship include the acute pharmacological effects of alcohol, autonomic hyperactivity associated with a chronic state of withdrawal, possible interactions between alcohol and medication, and the effects of alcohol on compliance with medication use and lifestyle recommendations [27].

Limitations

This study had some limitations. First, because the participants were limited to those who had undergone a specific health examination, selection bias may have occurred. Second, we did not collect data regarding factors related to hypertension, such as salt intake, cardio-ankle vascular index, carotid intima-media thickness, and creatinine levels; therefore, we were unable to adjust for these confounding factors. Third, we did not collect data regarding the blood relationships of the study participants and were unable to adjust for these confounding factors. Fourth, this study included older Japanese individuals who lived in a particular area; therefore, caution is needed when generalizing the results.

## Conclusions

The minor allele of the ACE gene rs4309 is a risk factor for hypertension in older Japanese community-dwelling individuals. Identification of high-risk individuals for hypertension based on the rs4309 genotype of the ACE gene may allow for the implementation of targeted lifestyle interventions from an early age, potentially preventing or delaying the onset of hypertension. 

## Appendices

**Table 4:** Characteristics of study participants according to ACE genotypes ACE, angiotensin-converting enzyme gene; BMI, body mass index; DBP, diastolic blood pressure; Q1, first quartile; Q3, third quartile; SBP, systolic blood pressure.

	CC (n=332)	CT (n=773)	TT (n=661)	P value
	Median (Q1-Q3)	Median (Q1-Q3)	Median (Q1-Q3)	
Age (years)	74.0 (69.3-80.0)	73.0 (69.0-79.0)	73.0 (69.0-79.0)	0.132
Height (cm)	154.9 (148.8-160.9)	153.6 (148.7-160.4)	154.1 (149.3-160.7)	0.561
Weight (kg)	54.3 (48.0-62.8)	53.8 (47.9-61.8)	53.8 (47.8-61.3)	0.836
BMI (kg/m^2^)	22.9 (20.8-25.0)	22.7 (20.6-24.9)	22.7 (20.6-25.0)	0.745
SBP (mmHg)	137.0 (126.0-150.0)	136.0 (124.0-149.0)	137.0 (125.0-149.0)	0.631
DBP (mmHg)	76.0 (68.3-85.0)	75.0 (68.0-84.0)	76.0 (68.0-84.0)	0.51
	n (%)	n (%)	n (%)	
Hypertension	234 (70.5)	556 (71.9)	439 (66.4)	0.072
Antihypertensive medication use	168 (50.6)	403 (52.1)	296 (44.8)	0.018
Sex (male)	130 (39.2)	283 (36.6)	240 (36.3)	0.654
Excessive alcohol consumption	6 (1.8)	18 (2.3)	16 (2.4)	0.818
Current smoking	16 (4.8)	55 (7.1)	56 (8.5)	0.109
Exercise	158 (47.6)	412 (53.3)	338 (51.1)	0.216

**Table 5:** Multivariable analysis results according to ACE genotypes ACE, angiotensin-converting enzyme gene; BMI, body mass index; CI, confidence interval; OR, odds ratio.

	Unit	Crude	Model 1	Model 2
		OR (95% CI)	OR (95% CI)	OR (95% CI)
ACE genotype				
TT	Reference	1	1	1
CT	Reference	1.296 (1.035-1.623)	1.301 (1.028-1.647)	1.301 (1.028-1.648)
CC	Reference	1.207 (0.907-1.607)	1.128 (0.837-1.521)	1.122 (0.831-1.515)
Age	+1 year		1.073 (1.055-1.092)	1.075 (1.056-1.094)
BMI	+1 kg/m²		1.178 (1.137-1.220)	1.176 (1.135-1.219)
Sex	Female/male		1.032 (0.826-1.289)	1.022 (0.808-1.294)
Excessive alcohol consumption	Yes/no			2.343 (1.042-5.266)
Current smoking	Yes/no			0.853 (0.562-1.297)
Exercise	Yes/no			0.918 (0.741-1.137)

**Table 6:** Multivariable analysis results according to ACE genotypes in men ACE, angiotensin-converting enzyme gene; BMI, body mass index; CI, confidence interval; OR, odds ratio.

	Unit	Crude	Model 1	Model 2
		OR (95% CI)	OR (95% CI)	OR (95% CI)
ACE genotype				
TT	Reference	1	1	1
CC and CT	Reference	1.230 (0.869-1.742)	1.264 (0.883-1.809)	1.265 (0.881-1.815)
Age	+1 year		1.035 (1.007-1.064)	1.038 (1.009-1.068)
BMI	+1 kg/m²		1.197 (1.123-1.277)	1.190 (1.115-1.269)
Excessive alcohol consumption	Yes/no			3.694 (1.064-12.818)
Current smoking	Yes/no			0.767 (0.482-1.218)
Exercise	Yes/no			0.797 (0.558-1.137)

**Table 7:** Multivariable analysis results according to ACE genotypes in women ACE, angiotensin-converting enzyme gene; BMI, body mass index; CI, confidence interval; OR, odds ratio.

	Unit	Crude	Model 1	Model 2
		OR (95% CI)	OR (95% CI)	OR (95% CI)
ACE genotype				
TT	Reference	1	1	1
CC and CT	Reference	1.288 (0.994-1.669)	1.219 (0.926-1.605)	1.220 (0.927-1.606)
Age	+1 year		1.099 (1.074-1.125)	1.100 (1.074-1.125)
BMI	+1 kg/m²		1.167 (1.118-1.217)	1.167 (1.118-1.218)
Excessive alcohol consumption	Yes/no			1.199 (0.376-3.823)
Current smoking	Yes/no			1.033 (0.370-2.887)
Exercise	Yes/no			1.022 (0.780-1.338)
